# Optimization of Sequential Enzymatic Hydrolysis in Porcine Blood and the Influence on Peptide Profile and Bioactivity of Prepared Hydrolysates

**DOI:** 10.3390/ijms26083583

**Published:** 2025-04-10

**Authors:** Cristina Moreno-Mariscal, Federico Moroni, Jaume Pérez-Sánchez, Leticia Mora, Fidel Toldrá

**Affiliations:** 1 Instituto de Agroquímica y Tecnología de Alimentos (IATA-CSIC), Avenue Agustín Escardino 7, 46980 Paterna, Spain; cmormar@iata.csic.es (C.M.-M.); ftoldra@iata.csic.es (F.T.); 2 Instituto de Acuicultura Torre de la Sal (IATS-CSI), 12595 Ribera de Cabanes, Spain; federico.moroni@csic.es (F.M.); jaime.perez.sanchez@csic.es (J.P.-S.)

**Keywords:** porcine blood, enzymatic hydrolysis, bioactive peptides, antioxidant, hypoglycemic, anti-inflammatory

## Abstract

The search for new alternatives for the revalorization of porcine blood is crucial due to the large quantities that are annually generated in slaughterhouses. In this study, a sequential enzymatic hydrolysis of pig blood was optimized using different combinations of the enzymes, namely, Alcalase 4.0 L and Protana™ Prime, Flavourzyme 1000 L, and Protamex^®^, as a sustainable method for obtaining extracts rich in bioactive peptides. All the assayed hydrolysates exhibited different peptide profiles and showed in vitro antioxidant, hypoglycemic, and anti-inflammatory activity, although their values differed significantly depending on the type of hydrolysis in ABTS, FRAP, and ORAC assays, as well as in the determination of the inhibitory activity of DPP-IV, NEP, TACE, and MGL enzymes. The hydrolysate obtained by the combination of Alcalase 4.0 L, Flavourzyme 1000 L, and Protana™ Prime (AFPP) resulted in the highest hydrolysis degree (33.39 ± 0.98%), and its peptide profile reflected a higher amount of peptides < 3 kDa. This hydrolysate also obtained significantly higher values for ABTS and the inhibition of TACE and MGL. However, APP2 stood out in NEP inhibition (79.39 ± 3.91%), while APPP was notable for DPP-IV inhibition (43.02 ± 1.39%). The analysis of the hydrolysates using mass spectrometry in tandem allowed for the identification of those sequences that are potentially responsible for the biological activities determined, which were characterized using in silico bioinformatic tools. The results show the potential of using sequential enzymatic hydrolysis in porcine blood to obtain multifunctional peptides.

## 1. Introduction

The meat industry generates a large amount of by-products that can represent a significant environmental problem if not managed properly [1]. According to the European Food Safety Agency, animal by-products (ABPs) are materials obtained from animals that are not intended for human consumption, including slaughterhouse waste such as skin, bones, blood, fat, or offal [2]. In the European Union (EU), 20 million tonnes of these ABPs are generated annually, making their utilization a challenge [2]. In particular, the latest available data show that 245 million pigs were slaughtered in the EU in 2020 [3]. Considering that approximately 4% of the animal live weight is blood, the search for new alternatives for the revalorization of this by-product is crucial to take advantage of its high nutritional value due to its high protein content [4].

In recent years, among the several technologies developed, enzymatic hydrolysis represents one of the most important alternatives for the revalorization of ABPs, specifically pig blood, as it is an economical and sustainable method that does not generate any waste after its application [5]. Enzymatic hydrolysis is a biotechnological process that allows for the fragmentation of proteins into peptides and amino acids using specific enzymes [6]. This process improves the functionality of food products and allows for the obtention of bioactive peptides, which are fragments between 2 and 20 amino acids that have been shown to have antioxidant, antimicrobial, hypoglycemic, antihypertensive, or anti-inflammatory properties, among others [7,8,9,10,11]. Regarding ABPs from porcine slaughter, hydrolysates obtained from its by- and co-products, such as heart, liver, lung, spleen, or kidney, have recently been reported to have antioxidant properties [12]. Indeed, Hwang et al. [12] has demonstrated the protection of the tripeptide WYR to oxidative stress identified in porcine liver hydrolysate tested in *Caenorhabditis elegans* model [13]. López-Pedrouso et al. [14] also reported the potential of porcine liver to obtain antihypertensive peptides through the inhibition of the angiotensin converting enzyme-I (ACE-I). Moreover, ABPs from other species like the skin from sheep or camel also showed the potential to exert antidiabetic activity via the inhibition of dipeptidyl peptidase-IV (DPP-IV) when they are hydrolyzed [15]. The positive effects reported from peptides present in poultry by-product hydrolysates include lipid regulation, antioxidant and antihypertensive activities, and effects on the liver as reported via in vitro and cell culture tests and in vivo studies [16]. Additionally, fish by-products such as heads, bones, scales, or viscera are also acquiring special relevance in recent years. As an example, the dipeptide CP obtained from a hydrolysate of *Chromis notata* was reported as a potent anti-oxidant and antimicrobial, and it also showed effects against atopic dermatitis [17].

Sequential hydrolysis is a strategy that optimizes the release of these compounds by using enzymes in successive stages, which also improves the efficiency of the hydrolysis process [18]. Therefore, the choice of the number of enzymes and the type of protease activity has a direct impact on the type of generated peptides and, consequently, their potential biological activity [19]. Thus, bioactive peptides may have different applications in the food industry as functional foods or in the development of new active packaging, in the formulation of animal feed to improve its nutritional value, and in the pharmaceutical industry as components with preventive or therapeutic potential [20,21,22].

The purpose of this study was to optimize the sequential hydrolysis of porcine blood using different enzyme combinations of endoproteases Alcalase 4.0 L and Protamex^®^, and exoproteases Protana™ Prime or Flavourzyme, with both endo- and exoprotease activities, evaluating their impact on antioxidant, hypoglycemic, and anti-inflammatory biological activities in vitro, as well as on the peptide profile using mass spectrometry techniques and in silico bioinformatics tools.

## 2. Results and Discussion

### 2.1. Degree of Hydrolysis and Molecular Size Profile of Generated Peptides of Porcine Blood Hydrolysates

**Figure 1:**
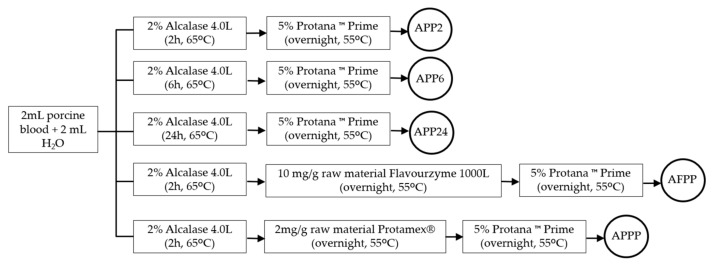
Scheme of porcine blood hydrolysates preparation.

The degree of hydrolysis (DH), understood as the amount of peptide bonds released during the hydrolysis process, is a crucial parameter by which to evaluate the efficiency of an enzymatic hydrolysis process [23]. As can be seen in Figure 1, the hydrolysis of porcine blood was performed using different enzyme combinations and hydrolysis periods to determine its impact on peptides generation.

**Figure 2:**
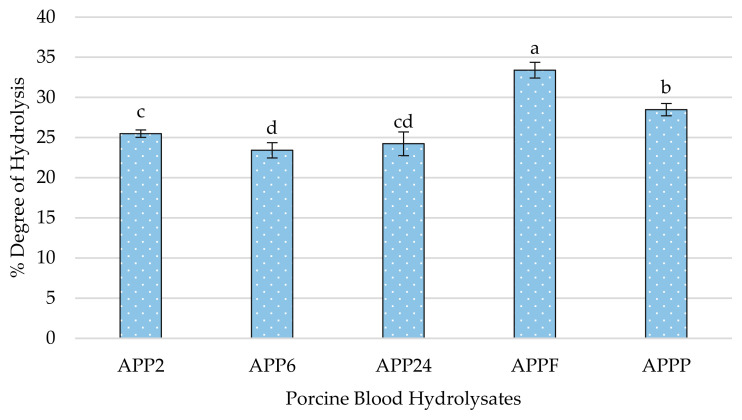
Degree of hydrolysis calculated in the porcine blood hydrolysates. Values were obtained via the mean of three replicates. Bars with different letters show significant differences between samples (*p* < 0.05).

The results (Figure 2) showed significant differences between treatments, with hydrolysis values ranging from 23.42% to 33.39%. The AFPP hydrolysate, obtained by sequential hydrolysis of Alcalase 4.0 L, Protana™ Prime, and Flavourzyme 1000 L, presented the highest DH value (33.39 ± 0.98%), followed by the APPP hydrolysate (Alcalase 4.0 L, Protana™ Prime, and Protamex^®^), with 28.48 ± 0.77%. Those hydrolysates, based on sequential hydrolysis with only two enzymes (Alcalase 4.0 L and Protana™ Prime), resulted in the lowest DH values. The statistical analysis showed that the type and the number of enzymes used for the hydrolysis significantly influenced the DH. The combination of three enzymes in AFPP resulted in the highest DH, suggesting that the inclusion of Flavourzyme 1000 L facilitated a larger release of peptide fragments due to its high proteolytic capacity with simultaneous endo- and exoprotease activity. Similarly, albeit to a lesser extent, the use of Protamex^®^ in APPP also facilitated larger peptide generation, but its endopeptidase activity was not as potent as that of Flavourzyme 1000 L [24].

Moreover, Carrera-Alvarado et al. [25] showed that the combination of several enzymes instead of one resulted in a significant increase in DH. In this study, chicken blood hydrolyzed with the combination of Alcalase 2.4 L, Flavourzyme^®^, and Protana UBoost^®^ showed the highest DH, with a rate of 30.29%, followed by the hydrolysates AP (Alcalase 2.4 L + Protana™ Prime), APP (Alcalase 2.4 L + Protana Prime™ + Protana UBoost^®^), and PF (Protana™ Prime + Flavourzyme^®^), with DH values between 25 and 30%. In addition, the longer hydrolysis time using alcalase does not necessarily lead to a higher degree of hydrolysis. In fact, APP6 (6 h) presented the lowest DH (23.42 ± 0.95%), while APP24 (24 h) only reached a DH of 24.23 ± 1.48%, with non-significant differences with APP2 (2 h).

These findings suggested that Alcalase 4.0 L is more effective in the first hours of the reaction. In fact, the results obtained by Bao et al. [26] showed that the hydrolysis of egg yolk with alcalase with 1.2% enzyme/substrate ratio (E/S) obtained during the first 60 min presented non-significant differences compared to values obtained after 150 min, indicating a maximum cleavage within the first 30 min. A similar behavior was observed in yellowfin tuna stomachs hydrolyzed with Alcalase 2.4 L at concentrations ranging from 0.2 to 3% (E/S). In all cases, a rapid initial increase in DH was observed during the first 90 min, but it became stationary after longer periods [27]. This may be explained by the decrease in peptide bond availability during the hydrolysis process, the competition between the accumulated peptides and proteins to be hydrolyzed, the reduction in the enzyme activity due to pH changes, or even some enzyme autolysis [28].

**Figure 3:**
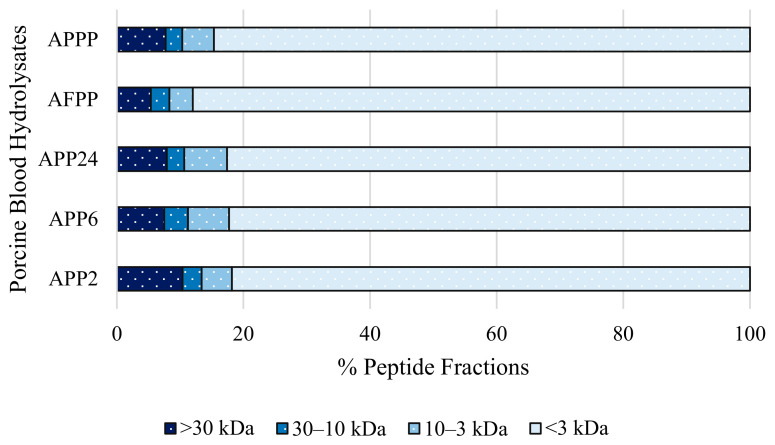
Molecular size distribution of different porcine blood hydrolysates obtained using membrane ultrafiltration.

As can be seen in Figure 3, the use of different enzymes not only influences the degree of hydrolysis but also the peptide profile of the hydrolysates. According to the peptide profile, a direct relationship between an increase in DH and a greater presence of peptides with a molecular size of less than 3 kDa can be observed. Following this trend, AFPP hydrolysate, which obtained the highest DH, also has the largest fraction, with peptides <3 kDa representing 87.98 ± 1.12% of the hydrolysate.

This can be explained by the combined action of Protana™ Prime and Flavourzyme 1000 L, both with intense exoprotease activity. In case of APPP, which includes two endoproteases (Alcalase 4.0 L and Protamex^®^) and only one exoprotease (Protana™ Prime), this fraction is lower than in AFPP, but the same trend is maintained as this hydrolysate has the second-highest degree of hydrolysis and, likewise, the second-largest fraction < 3 kDa, with 84.65 ± 0.67%. Regarding the other fractions, the highest >30 kDa fraction was observed in the APP2 hydrolysate (10.39 ± 0.15%), which may be related to the shorter hydrolysis time and the presence of only two enzymes. Hydrolysates of two enzymes, APP6 and APP24, showed similar profiles. Regarding 30–10 kDa and 10–3 kDa fractions, they were similar in all hydrolysates except in AFPP.

### 2.2. Antioxidant Capacity of Porcine Blood Hydrolysates

**Figure 4:**
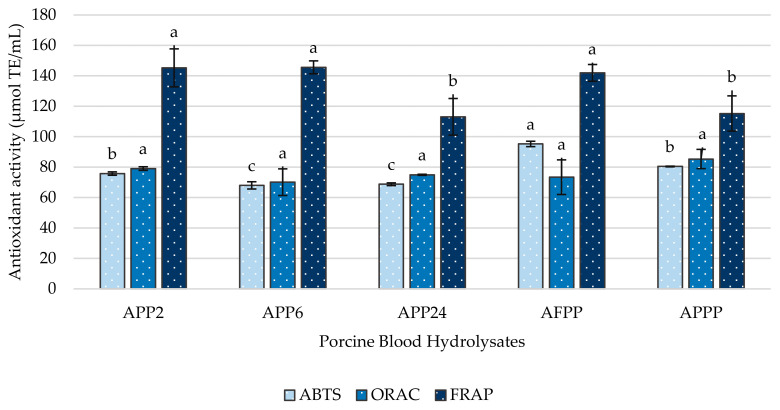
Antioxidant activity of porcine blood hydrolysates measured using ABTS, ORAC, and FRAP assays. Values were obtained via the mean of three replicates. Bars with different letters show significant differences between samples (*p* < 0.05).

The antioxidant activity of the different hydrolysates was determined by three different methods: ABTS, ORAC, and FRAP (Figure 4). Several conditions can significantly influence the antioxidant activity of peptides. One of the main factors is the molecular size, with small peptides potentially showing the highest bioactivity [29]. The impact of peptide size on radical scavenging capacity has frequently been proved. In fact, Cheung et al. [30] showed that the most bioactive fraction in the ABTS assay of hake hydrolysates had peptides smaller than 1.4 kDa. However, in ORAC and FRAP assays, this trend is not so clear. Antioxidant activity is not only related to the size of the peptides but also includes other properties such as the polarity, amino acid sequence, or hydrophobicity; even a higher presence of residues such as Gly, Ala, Leu, Trp, Tyr, Met, Arg, Phe, or Val has been related to a higher radical scavenging capacity [31,32,33].

As previously described, all hydrolysates showed around 80% of peptides with molecular size < 3 kDa. This intense hydrolysis would explain the similarities observed between antioxidant activities in hydrolysates. The AFPP hydrolysate showed the highest bioactivity in ABTS, with a value of 95.26 ± 1.78 µmol/mL, followed by APPP with 80.46 ± 0.25 µmol/mL, APP2 with 75.79 ± 1.10 µmol/mL, and APP24 and APP6 with non-significant differences between them.

These results followed the same trend as the results obtained in DH. In fact, Ozturk-Kerimoglu et al. [34] also showed that AP hydrolysate (Alcalase^®^ + Protana^®^ Prime) showed the highest DH and antioxidant activity values in ABTS in comparison to those obtained in A (Alcalase^®^) and AF (Alcalase^®^ + Flavourzyme^®^) hydrolysates of chicken feet. Thus, a higher degree of hydrolysis frequently indicates a higher presence of small peptides and, therefore, a higher biological antioxidant activity by ABTS assay. Finally, the hydrolysates did not show significant differences in the ORAC assay, showing antioxidant activity values between 70.12 and 85.35 µmol/mL. However, the APP24 and APPP hydrolysates, in which a more intense endoprotease activity took place, showed significantly lower values than the rest of the hydrolysates in the FRAP assay, with values around 20% lower.

### 2.3. Hypoglycaemic Potential of Porcine Blood Hydrolysates

The hypoglycemic activity of the hydrolysates was evaluated through the determination of the DPP-IV and NEP enzymes inhibition.

**Figure 5:**
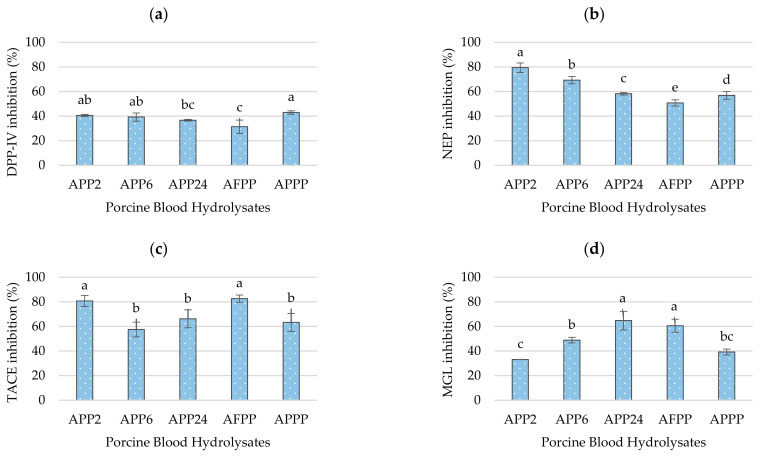
Bioactivities of porcine blood hydrolysates measured through the inhibition of different enzymes: (**a**) DPP-IV; (**b**) NEP; (**c**) TACE; (**d**) MGL. Values calculated as the average of three replicates. Bars labeled with different letters indicate significant differences between samples (*p* < 0.05).

The enzyme DPP-IV is a serine protease that plays a key role in metabolism, primarily by degrading incretin hormones such as glucose-dependent insulinotropic polypeptide (GIP) and glucagon-like peptide (GLP-1) [35]. By inactivating incretins, DPP-IV reduces insulin secretion, which has a direct impact on blood glucose levels. Therefore, the inhibition of this enzyme has recently been studied in clinical assays, enabling the development of treatments of diseases like type 2 diabetes [36]. Porcine blood hydrolysates showed DPP-IV inhibition values between 31.42 ± 5.43% and 43.02 ± 1.39% (Figure 5a), with APPP being the highest. However, APPP did not show significant differences with respect to APP2 (40.58 ± 0.87%) and APP6 (39.29 ± 3.33%), and these, in turn, did not show significant differences with respect to APP24 (36.71 ± 0.66%). Thus, the inhibition of the DPP-IV enzyme is not only related to the size of the peptides or the degree of hydrolysis but also to the type of peptidase used and, consequently, the amino acids composition of these peptides [37]. Thus, Carrera-Alvarado et al. [38] reported inhibition values of 24.99% in cured-ham bone samples hydrolyzed with Alcalase^®^ and Protana^®^ Prime pretreated with pepsin, showing that sequential hydrolysis increased the presence of DPP-IV inhibitory peptides. In addition, other authors have reported DPP-IV inhibitory results for other hydrolysates from meat by-products such as cow skin discards [39], egg by-products such as eggshell membrane [40], or even fish by-products such as tilapia skin [41] and salmon by-products, obtaining positive results in Caco-2 cell models [42].

Neprilysin (NEP) is a zinc-dependent endopeptidase widely distributed in several tissues of the human body and responsible for the degradation of peptides such as bradykinin, substance P, and natriuretic peptides [43]. Its inhibition has been extensively studied for its crucial role in the regulation of blood pressure in pathologies such as heart failure [44]. However, NEP has also been shown to inactivate GLP-1, so its inhibition also plays an important role in blood glucose homeostasis and may even be involved in metabolic processes that trigger overweight and diabetes [45]. The NEP inhibitory activity of hydrolysates is shown in Figure 5b. The results show significant differences (*p* < 0.05) between all hydrolysates; therefore, both enzyme selection and hydrolysis times (2, 6, or 24 h) play an important role in obtaining NEP inhibitory peptides. Thus, the APP2 hydrolysate showed the highest percentage of NEP inhibition, with a value of 79.39 ± 3.91%, followed by APP6 (69.29 ± 2.98%) and APP24 (58.20 ± 0.96%). Hydrolysates APPP and AFPP showed the lowest inhibition values (56.92 ± 3.15 and 50.76 ± 2.51%, respectively). The mechanism of action and the type of peptides obtained through enzymatic hydrolysis that inhibit NEP is still unknown; however, these results agree that higher hydrolysis values decrease the NEP inhibitory capacity of the hydrolysates. Furthermore, it can be seen how the use of endoproteases such as Protamex^®^, compared to enzymes with exoprotease activity such as Flavourzyme 1000 L, might favor the presence of these NEP inhibitory peptides. Some recent studies have reported in vitro NEP inhibition data on peptides from dry-cured ham, chicken carcass hydrolysates, and beef liver hydrolysates [46,47,48].

### 2.4. Anti-Inflammatory Potential of Porcine Blood Hydrolysates

The anti-inflammatory activity was studied by analyzing the TACE-inhibitory and MGL-inhibitory activity of the pig blood hydrolysates. The enzyme TACE (tumor necrosis α-converting enzyme) or ADAM17 is a membrane protease whose main function is the release of several cell surface proteins such as cytokines, receptors, or growth factors, which has a fundamental role in cell communication and in the regulation of the organism biological responses [49]. One of its most important substrates is tumor necrosis factor-alpha (TNF-α), a pro-inflammatory cytokine that is essential in the immune response and chronic inflammatory processes [50]. Due to its involvement in various pathological processes, such as arthritis, cardiovascular, and even oncological disorders, the inhibition of this enzyme is of great therapeutic interest [51]. The hydrolysates showed TACE-inhibitory values between 57.5 and 82.6% (Figure 5c), with AFPP hydrolysate having the highest inhibitory value. In this case, no clear relationship can be established between TACE inhibition and the degree of hydrolysis; however, the choice of enzymes and the duration of hydrolysis could be a key factor. Udechukwu et al. [52] reported, for the first time, the presence of TACE inhibitory peptides in food sources through the identification of four tripeptides (CQV, QCV, QVC, QCA), with inhibition rates higher than 70% in grain rye secalin. These peptides showed a dose-dependent inhibitory activity, with 5 µM showing the highest inhibition. Subsequently, Heres et al. [47] reported that dipeptide ES at a concentration of 1 mM exerted 50% TACE inhibition.

On the other hand, monoacylglycerol lipase (MGL) is a key enzyme in lipid metabolism whose main function is the degradation of 2-arachidonoylglycerol (2-AG), an endocannabinoid with effects on the immune system and the nervous system [53]. Its role in inflammation is crucial as its activity generates arachidonic acid, which is a precursor of pro-inflammatory eicosanoids. Thus, in diseases such as cancer, neurodegenerative processes, and metabolic disorders, increased MGL activity has been linked to increased inflammation and progression of these pathologies [54]. In this case, all blood hydrolysates also showed inhibition against the MGL enzyme (Figure 5d). APP24 and AFPP hydrolysates obtained the highest MGL inhibitory values, i.e., 64.76 ± 7.54% and 60.56 ± 5.35%, respectively. Hayes et al. [55] recently reported 63.19% MGL inhibition of peptides, with a molecular size < 3 kDa obtained from hydrolysates of *Maurolicus muelleri* with Alcalase^®^. This study provides a new source of research on natural peptides with anti-inflammatory potential and dual inhibition of TACE and MGL.

### 2.5. Free Amino Acid Profile of Porcine Blood Hydrolysates (FAAs)

Table 1 presents the free amino acid content of the hydrolysates. The AFPP hydrolysate showed the highest amount of free amino acids, with a value of 74.286 ± 1.113 mg/mL, followed by APPP (59.628 ± 0.205 mg/mL) and APP2 (52.291 ± 0.110 mg/mL).

Statistical analysis showed significant differences (*p* < 0.05) among all samples such that the type of enzymatic treatment significantly influences the concentration of free amino acids. In fact, longer hydrolysis times do not result in a higher presence of free amino acids as this enzyme only exerts endoprotease activity. In fact, amino acids, namley, Asp, Glu, Ser, Gly, His, Thr, Ala, Val, Leu, Phe, and Lys, showed significantly higher values (*p* < 0.05) in APP2 than APP6 and APP24. On the other hand, the combination of Alcalase 4.0 L, Protana™ Prime, and an additional enzyme (Flavourzyme 1000 L or Protamex^®^) had a positive effect on the release of amino acids. However, despite AFPP showing the highest amount of amino acids, the most abundant amino acids were the same in all hydrolysates, i.e., Leu, Lys, Val, Ala, Phe, and His, with most of them being essential amino acids. In addition, the presence of βAla, γaba, and Orn was detected. These results are in agreement with previous studies where the use of Flavourzyme in pig liver hydrolysates allowed for a higher yield of free amino acids compared to other endopeptidases [56]. In addition to the dual endo- and exoprotease activity of Flavourzyme 1000 L, the use of Protana™ Prime, which has a very intense exoprotease activity, also explains the high proportion of free amino acids in the hydrolysates. Dominguez et al. [57] reported a higher release of free amino acids using Protana^®^ Prime than Alcalase 2.4 L in trout viscera hydrolysates, with a combination of both enzymes being the best option to generate FAAs.

### 2.6. Peptidomics and In Silico Analysis of Identified Peptides from Porcine Blood Hydrolysates

**Figure 6:**
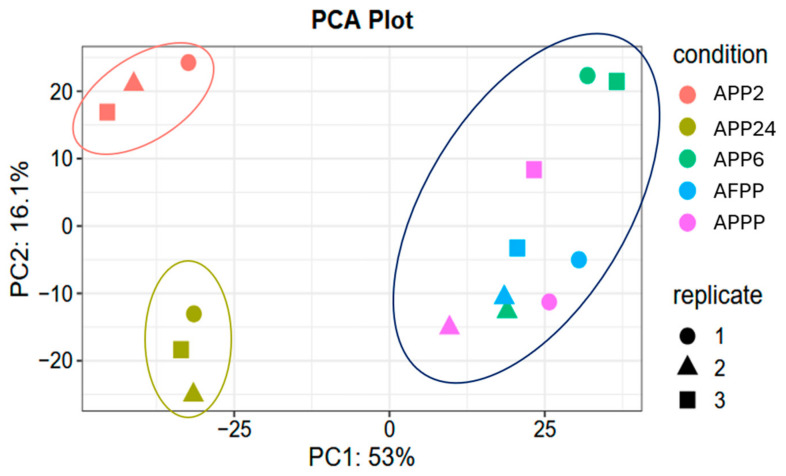
Principal component analysis (PCA) plot of identified peptides in porcine blood hydrolysates. Samples with the same color are replicates (with different forms) of the same hydrolysate.

All hydrolysates were analyzed by LC-MS/MS for characterization of the peptides present in each hydrolysate. A total of 539 peptide sequences were identified in the APP2 hydrolysate: 142 in APP6, 241 in APP24, 100 in AFPP, and 124 in APPP. These peptides came from 675 pattern proteins, and 311 exhibited significant differences between samples. In this context, Figure 6 presents the principal component analysis (PCA) plot of the peptides of the processed samples. The results revealed three different clusters, with principal component 1 accounting for 53% of the observed variance, while principal component 2 explained 16.1%.

The identified peptide sequences were subsequently analyzed using in silico tools, as showed in Table 2. The peptides were between 7 and 37 residues long. Regarding the bioactive potential of the identified peptides, the APP2 hydrolysate showed the highest number of sequences, with a peptide ranker ratio above 0.75, with the DRPFPDF and QPFPIRP sequences being above 0.9. Also, the sequences GYFVFGPTGCNLEGFF (APP6), AAEGACQQCSCRPMWCLTCMGKWFASRQDPQRPDTWL, and QPFPIRP (APP24) showed PRR values above 0.9. Regarding the ability of the peptides to produce possible allergic reactions, seven sequences were found to be possible allergens in APP2; APP24 and APPP had four; while AFPP and APP6 had only two out of the total number of peptides, with a peptide ranker ratio (PRR) > 0.75.

Hydrophobicity and net charge are also shown in Table 2. Antioxidant activity is known to be highly dependent on the hydrophobicity of the peptide, as well as the amino acids composition [7]. Thus, the peptides LFPNPPPPPPPQP (APP2), GPGGPGTWKPGRPEPG (APP2), and YQEPVLGPVRGPFPIIV (present in all hydrolysates) showed the highest free radical scavenging capacity (FRS) scores and could exert antioxidant potential through scavenging activity and metal ion chelation. Similarly, the peptides with the highest antioxidant potential according to their chelating capacity were ADPVNFPKL (APP2) and DRPFPDF (APP2). In contrast, the inhibitory activity of DPP-IV is affected by the presence of Pro or Ala in the second residue of N-terminal [58,59]. As previously mentioned, the characteristics of the peptides with inhibitory capacity of NEP, TACE, or MGL enzymes have not been extensively studied yet. However, a recent study revealed that the presence of certain amino acids, as well as their position at the C- or N-terminus, can lead to an increase in NEP inhibitory activity. Thus, higher NEP inhibition values were observed with the presence of Arg or Glu in the C-terminal position and Ala in the N-terminal position, with the dipeptide VV obtaining the highest NEP inhibition value (75%) at 1 mM [46].

Subsequently, peptides with a PRR value higher than 0.75 were subjected to simulated gastrointestinal digestion using bioinformatics tools with pepsin, trypsin, and chymotrypsin enzymes, as shown in Table 3. Most of the peptides listed in Table 2 would not be able to cross the intestinal barrier intact due to their size, but many of them would be hydrolyzed into small peptide fragments with bioactive potential, as shown in Table 3. Some of the generated fragments have been previously reported as inhibitors of ACE (GK), DPP-IV (GPF), or NEP-2 (VF) or antioxidants (PK); whereas others, such as DR, PF, GY, or V, exhibit multiple bioactivities that may exert a multifunctional role in the organism. The cell penetration probability (CPP) of the active fragments increased with respect to the initial peptides. Thus, the PR, PK, and GK dipeptides also showed the highest probability of cell penetration (CPP). The dipeptide PR released from the DGPLPRP and VDGPLPRP peptide sequences had the highest CPP value (0.8096), followed by GK (0.5298), present in SMGKGYYLKGKIGKVPVRF, and PK (0.5177), obtained from ADPVNFPKL. Consequently, these fragments may have a higher probability to exert their bioactivity.

## 3. Materials and Methods

### 3.1. Chemicals and Reagents

Alcalase 4.0 L and Protana™ Prime, Flavourzyme 1000 L, and Protamex^®^ were purchased from Novozymes A/S (Bagsvaerd, Denmark), human neprilysin protein tag free from Acro BioSystems (Basel, Switzerland), and aminopeptidase M from Merck (Darmstadt, Germany). Thiorphan, N-Succinyl-Ala-Ala-Phe-7-amido-4-methylcoumarin, HEPES, o-phthaldialdehyde (OPA), ferric chloride, potassium ferricyanide, L-tryptophan, 2,2-azino-bis(3-ethylbenzothiazoline-6-sulfonic acid) diammonium salt (ABTS), ascorbic acid, (±)-6-hydroxy-2,5,7,8-tetramethylchromane-2 carboxylic acid (Trolox), 2,2-azobis(2-methylpropionamidine) dihydrochloride (AAPH), and fluorescein were purchased from Sigma-Aldrich, Co. (St. Louis, MO, USA). Butylated hydroxytoluene (BHT) and potassium persulfate were supplied from Panreac Química S.A.U. (Barcelona, Spain). All other reagents utilized in the assays were of analytical-grade quality.

### 3.2. Preparation of Samples and Enzymatic Hydrolysis

Fresh porcine blood was subjected to an ultrasound pretreatment for 1 h at 35 kHz, and 2 mL of blood was diluted with 2 mL of bidistilled water. The hydrolysis process was performed using sequential enzymatic hydrolysis according to the blood protein content (21%) previously determined using the Dumas method [60].

First, 2% Alcalase 4.0 L was added to all hydrolysates and incubated at 65 °C at different times: 2 h (APP2, AFPP, APPP), 6 h (APP6), or 24 h (APP24). Next, different enzymes were applied as follows: 5% Protana™ Prime (APP2, APP6, APP24), 10 mg/g raw material Flavourzyme 1000 L (AFPP), and 2 mg/g raw material Protamex^®^ (APPP) were used for an overnight treatment at 55 °C. Finally, a second addition of 5% Protana™ Prime was applied overnight at 55 °C to the AFPP and APPP hydrolysates. The percentage of the added enzymes was determined as recommended by the manufacturer. Finally, the hydrolysates were heated in a water bath (85 °C, 15 min) for enzyme inactivation. All the hydrolysates were performed in triplicate and stored at −20 °C.

### 3.3. Degree of Hydrolysis

The hydrolysis degree (DH) of porcine blood hydrolysates was calculated as described by Nielsen et al. [22], with certain modifications. Briefly, 36 µL of samples and controls, at a concentration of 2 mg/mL, were mixed with 270 µL of OPA reagent in a transparent 96-well plate. L-serine was used as positive control (0.9516 meq/L). The absorbance was recorded at 340 nm following a 2 min incubation using a CLARIOstar^®^ multimode microplate reader (BMG LABTECH, Ortenberg, Germany). DH values were calculated as follows:

(i) “h” determination:

(1) $$Serine-NH2=\frac{OD\;sample-OD\;blank}{OD\;standard-OD\;blank}\;x\;0.9515\frac{meqv}{L}\;x\;0.1\;x\;\frac{100}{\;X\;}\;x\;P$$

where Serine-NH2 = meqv serine NH2/g protein; X = g sample; P = protein % in sample; and 0.1 is the volume of sample per liter (L).

(2) h=SerineNH2−β/α(meqv/g protein)

where α and β are specific constants depending on the raw material (α = 1.00; β = 0.4).

(ii) DH calculation:

(3) $$DH\left(\%\right)=\frac{h}{htot}\;x\;100$$

where htot is also a specific values depending on the raw material (htot = 8.8).

### 3.4. Ultrafiltration

A volume of 500 µL of each hydrolysate was fractionated (in triplicate) using 3 different Amicon^®^ ultra 0.5 mL filters (Merck Millipore Ltd., Cork, Ireland) with membranes with molecular weight cut-offs of 30, 10, and 3 kDa. The resulting fractions (>30 kDa, 30–10 kDa, 10–3 kDa, and <3 kDa) were freeze-dried and weighed to determine the peptide distribution based on its molecular weight.

### 3.5. Free Amino Acid Composition

The determination of free amino acids (FAAs) content was determined according to the method described by Flores et al. [61]. First, the sample was prepared by diluting the spray-dried hydrolysate with bidistilled water to obtain the appropriate concentration, considering an internal standard solution of 5 mM norleucine. The chromatographic separation of derivatized amino acids was performed using a reverse-phase HPLC chromatograph equipped with a Waters Pico Tag^®^ C18 column (3.9 × 300 mm; Waters Corp., Milford, MA, USA), with a flow rate of 1 mL/min at 52 °C. Elution was monitored at a wavelength of 254 nm. Phase A was prepared using a 0.07 M sodium acetate solution containing 2.5% (*v*/*v*) acetonitrile, adjusted to pH 6.55. For phase B, a mixture of acetonitrile–water–methanol in a 45:40:15 ratio was used. The quantification was performed using the response factors determined for each amino acid in the mixed standard series. The results were expressed as mg FAAs/mL sample.

### 3.6. Biological Activities

#### 3.6.1. Antioxidant Activities

##### ABTS Radical Scavenging Capacity

The ABTS assay was performed as described in Gallego et al. [62]. Briefly, a 7 mM ABTS stock solution was prepared with 2.45 mM potassium persulfate and incubated in darkness for 16 h to produce ABTS+. This solution was diluted with 50 mM PBS (pH 7.4) to achieve an absorbance of 0.70 ± 0.02 (working solution) and measured at 734 nm in a UV-visible spectrophotometer (Cary 60, Agilent Technologies, CA, USA) after 6 min of darkness incubation. Before the absorbance measure, 10 µL of each hydrolysate (10 mg/mL) was mixed with 990 µL of working solution. Results were calculated using a Trolox standard curve (µmol TE/mL), and ascorbic acid was used as the positive control.

##### Oxygen Radical Absorbance Capacity Assay (ORAC)

The ORAC assay was also carried out according to Gallego et al. [62]. In summary, 140 µL of each hydrolysate (50 µg/mL, prepared in triplicate) was incubated at 37 °C for 15 min with 70 µL of 0.2 mM fluorescein, which had previously been prepared in a 75 mM phosphate buffer (pH 7.4). Subsequently, 70 µL of 80 mM AAPH was added, and fluorescence was recorded at 1 min intervals over 100 min using excitation and emission wavelengths of 485 nm and 538 nm, respectively, in a CLARIOstar^®^ multimode microplate reader (BMG LABTECH, Ortenberg, Germany). Tryptophan served as the positive control, and the results are expressed as µmol TE/mL hydrolysate.

##### Ferric-Reducing Antioxidant Power (FRAP)

The reducing power assay was based on the method described by Huang et al. [63], with slight modifications. A total volume of 70 µL of each hydrolysate, in triplicate (20 mg/mL), was mixed with 70 µL of 0.2 M of phosphate buffer (pH 6.6) and 70 µL of potassium ferricyanide (1%), and this mixture was incubated at 50 °C for 20 min. After that, 70 µL of trichloroacetic acid (10%) was added and then centrifugated at 200× *g* for 10 min. Then, 140 µL of supernatant was collected and transferred into a 96-wellplate, and 140 µL of bidistilled water and 28 µL of ferric chloride (0.1%) were also added to each well. The absorbance was measured after 10 min of incubation at 690 nm using a CLARIOstar^®^ multimode microplate reader (BMG LABTECH, Ortenberg, Germany). BHT (2 mg/mL) served as the positive control, and results are shown as µmol TE/mL hydrolysate.

#### 3.6.2. Dipeptidyl Peptidase IV (DPP-IV) Inhibitory Activity

DPP-IV inhibition was determined using “DPP-IV inhibitor screening kit” (MAK203) (Sigma-Aldrich, Saint Louis, MO, USA). A total of 50 µL of each sample (20 mg/mL, in triplicate) was combined with 50 µL of enzyme solution in a black 96-well plate with a clear bottom and incubated at 37 °C for 10 min, protected from light. Then, 25 µL of substrate solution was added to each well. The fluorescence was measured (Ex/Em = 360/460 nm) in a CLARIOstar microplate reader (BMG Labtech, Ortenberg, Germany) in a kinetic mode for 30 min at 37 °C. Sitagliptin was used as the positive control, and bidistilled water as the negative control. The percentage of inhibition was calculated as follows:

(4) $$Slope=\frac{FLU2-FLU1}{T2-T1}=\Delta FLU/minute$$

(5) $$\%Inhibition=\frac{Slope\;EC-Slope\;SM}{Slope\;EC}\;x\;100\;$$

where Slope EC = the slope of the enzyme control; Slope SM = the slope of the sample inhibitor.

#### 3.6.3. Neprilysin (NEP) Inhibitory Activity

NEP inhibitory activity was assayed based on the methodology outlined by Moreno-Mariscal et al. [28]. The assay was performed by adding 25 µL of sample, in triplicate (20 mg/mL), and 25 µL of enzyme solution in a black 96-wellplate with clear bottoms. The enzyme solution was prepared by diluting the NEP stock solution (400 µg/mL in 0.1% BSA) 1:1000 with bidistilled water. The substrate solution consisted of the mixture of 0.2 mM N-Succinyl-Ala-Ala-Phe-7-amido-4-methylcoumarin (AMC) dissolved in a 50 mM HEPES/NaOH buffer (pH 7.4) and 0.75 µg/well of aminopeptidase M. Thiorphan (1 mM) was used as the positive control and bidistilled water as the negative. Fluorescence was then measured at excitation/emission wavelengths of 320/420 nm for 60 min at 37 °C using a CLARIOstar microplate reader (BMG Labtech, Germany). The percentage of inhibition was determined with the following equations, selecting two time points within the linear range.

(6) Δ Fluorescence (F)=T2−T1

(7) $$\%Inhibition=\frac{\Delta F\;negative\;control-\Delta F\;sample}{\Delta F\;negative\;control}\;x\;100$$

#### 3.6.4. Tumor Necrosis α-Converting Enzyme (TACE) Inhibitory Activity

TACE inhibition was determined using the ‘TACE Inhibitor Screening Assay Kit’ (Sigma-Aldrich, Saint Louis, MO, USA). First, 25 µL of sample (100 mg/mL, in triplicate) was combined with 50 µL of enzyme solution in a black 96-wellplate and incubated at 37 °C for 5 min. Then, 25 µL of substrate solution was added to each well. GM6001 was used as the positive control, and the fluorescence was measured at Ex/Em = 318/449 nm for 30 min using a CLARIOstar microplate reader (BMG Labtech, Germany). The percentage of inhibition was calculated as follows:

(8) $$Slope=\frac{FLU2-FLU1}{T2-T1}=\Delta \;FLU/minute$$

(9) $$\%Inhibition=\frac{Slope\;EC-Slope\;SM}{Slope\;EC}\;x\;100$$

where Slope EC = the slope of the enzyme control; Slope SM = the slope of the sample inhibitor.

#### 3.6.5. Monoacylglycerol Lipase (MGL) Inhibitory Activity

MGL inhibition assay was carried out by using the “Monoacylglycerol lipase Inhibitor Screening kit” (ab283388, Abcam, Cambridge, UK). A volume of 5 µL of samples, in triplicate (20 mg/mL), were mixed with 90 µL of enzyme solution in a black 96-wellplate with clear bottoms and incubated at 37 °C for 20 min. Then, 5 µL of substrate solution was added to each well. JJKK-048 was used as positive control, and the fluorescence was measured at Ex/Em = 360/340 nm for 60 min at 37 °C in a CLARIOstar microplate reader (BMG Labtech, Germany). The percentage of inhibition was calculated as follows.

(10) $$\frac{\Delta Fluorescence}{\Delta Time}=\frac{F2-F1}{T2-T1}$$

(11) $$\%Inhibition=\frac{Rate\;negative\;control-Rate\;sample}{Rate\;negative\;control}\;x\;100$$

### 3.7. Peptide Characterization

#### 3.7.1. Peptide Identification by Tandem Mass Spectrometry

A total of 190 ng of each sample, prepared in triplicate, was adjusted to a final volume of 20 µL using 0.1% FA and loaded into an Evotip Pure tip (EV2018, EvoSep, Denmark), following the manufacturer instructions. Tandem mass spectrometry (LC–MS/MS) analysis was conducted using a Tims TOF fleX mass spectrometer (Bruker, MA, USA). The sample within the Evotip Pure tip was eluted onto an analytical PepSep column (10 cm × 150 µm, 1.5 µm; Evosep, Denmark) using the Evosep One platform and analyzed with the 60 SPD chromatographic method specified by the manufacturer. The eluted peptides were ionized using a CaptiveSpray source at 1700 V and 200 °C and analyzed in ddaPASEF mode with the following TIMS parameters: custom mode; 1/K0 range of 0.67–1.29 V·s/cm^2^; ramp time of 100 ms; duty cycle at 100%; and a ramp rate of 9.42 Hz. The MS analysis was performed in PASEF scan mode with a mass range of 100–1700 m/z and positive ion polarity. MS/MS settings included four PASEF ramps, a total cycle time of 0.5 s, a charge range from 0 to 5, an intensity target of 12,500, and an intensity threshold of 1000, with active exclusion enabled. To allow for the selection of mono-charged peptides, the inclusion polygon was removed, enabling precursor selection across the full 1/K0 range (IMS). System sensitivity was verified using 50 ng of digested HELA proteins.

#### 3.7.2. Protein Analysis and Identification

Protein identification was conducted using FragPipe interface, which is a complete pipeline to analyze mass spectrometry-based proteomics data through the search engine MSFragger. A total of 4006 proteins were identified using the 30 SPD gradient. Thus, MSFragger searches were conducted to identify non-tryptic peptides. Database was generated on 2024/03/27 from Uniprot for Uniprot_SusScrofa. Differential expression analysis was conducted with Marker View 1.3 (Sciex). Peptide intensities were calculated via the MSFragger IonQuant algorithm and normalized by total sum. Principal component analysis (PCA), discriminant analysis (DA), and t-tests were conducted. Data analysis was conducted with the analyst package (http://fragpipe-analyst.nesvilab.org accessed on 27 March 2024) by FragPipe. The LFQ intensities were used with the parameters MaxLFQ Intensity, variance stabilizing normalization, and Perseus-type imputation. Principal component analysis (PCA) was determined in order to establish significant differences between samples.

#### 3.7.3. In Silico Analysis

##### Software Tools

The Peptide Ranker tool (http://distilldeep.ucd.ie/PeptideRanker/, accessed on 10 February 2025) was utilized to assess the potential bioactivity of the identified peptides, with scores nearing 1 indicating a higher probability of bioactivity. Then, the AnOxPePred 1.0 software (https://services.healthtech.dtu.dk/services/AnOxPePred-1.0/, accessed on 10 February 2025) was employed for antioxidant properties prediction based on chelating scores and free radical scavenging (FRS). To evaluate the allergenic potential of the peptides, the AllerTOP v.2.0 tool (https://www.ddg-pharmfac.net/allertop_test/, accessed on 10 February 2025) was applied. The hydrophobicity, net charge, and possible toxicity of the peptides were estimated with ToxinPred tool (http://crdd.osdd.net/raghava/toxinpred/, accessed on 10 February 2025) based on their amino acid composition. Finally, the CPPpred tool (http://bioware.ucd.ie/~compass/biowareweb/Server_pages/cpppred.php, accessed on 10 February 2025) was used to estimate the potential of the peptides to penetrate the cell (CPP), with scores closer to 1 indicating a greater probability of cellular penetration.

##### Simulated Gastrointestinal Digestion

Peptides with a peptide ranker ratio (PRR) beyond 0.75 were subjected to a simulated gastrointestinal digestion using BIOPEP database (https://biochemia.uwm.edu.pl/biopep/start_biopep.php, accessed on 10 February 2025). This digestion was carried out by selecting the enzymes pepsin (EC 3.4.23.1), trypsin (EC 3.4.21.4), and chymotrypsin (EC 3.4.21.1).

### 3.8. Statistical Analysis

All statistical analyses were conducted using RStudio v.4.3.3 software (R Foundation for Statistical Computing, Vienna, Austria). Data were evaluated using one-way ANOVA, followed by Tukey’s honest significant difference (HSD) test (*p*-value ≤ 0.05). The results are presented as the mean of replicates ± standard deviation.

## 4. Conclusions

The number and type of proteases used in the hydrolysis process significantly influence the peptide profile and, consequently, its bioactivity. The AFPP hydrolysate, obtained from the sequential hydrolysis of porcine blood using Alcalase 4.0 L, Flavourzyme 1000 L, and Protana™ Prime, exhibited the highest DH, the largest fraction of peptides < 3 kDa and FAAs, and significantly higher antioxidant activity determined by ABTS assay. The rest of hydrolysates also showed antioxidant and inhibitory activities of the enzymes DPP-IV, NEP, TACE, and MGL, evidencing their multifunctional character. However, APPP exhibited the highest DPP-IV inhibitory activity, while APP2 showed the greatest NEP inhibition. APP24 was the most effective against MGL, whereas AFPP displayed the highest TACE inhibition value. Peptides that could be responsible for these biological activities were identified by LC-MS/MS and characterized by in silico analysis. Simulated gastrointestinal digestion revealed that these peptides contained fragments whose bioactivity has previously been reported. These results confirmed that sequential enzymatic hydrolysis is a promising strategy for the revalorization of pig blood generating bioactive peptides. However, further studies are necessary to prove the viability of these hydrolysates for use as functional ingredients in human food or animal feed and as potential nutraceuticals. The synthesis of peptides that were shown to be potentially bioactive in silico and the development of in vivo tests, would help to confirm the bioactivity of the hydrolysates.

## Figures and Tables

**Table 1:** Free amino acid (FAAs) composition of porcine blood hydrolysates. The values presented are the means ± standard deviation of three replicates. Means in the same row without a common letter in superscript are significantly different (*p* < 0.05).

FAAs	Concentration (mg/mL of Hydrolysate)				
	APP2	APP6	APP24	AFPP	APPP
Aspartic Acid (Asp)	2.272 ± 0.001 ^c^	2.048 ± 0.007 ^d^	2.020 ± 0.000 ^d^	2.555 ± 0.036 ^a^	2.399 ± 0.007 ^b^
Glutamic Acid (Glu)	1.928 ± 0.001 ^b^	1.700 ± 0.005 ^c^	1.729 ± 0.001 ^c^	2.257 ± 0.033 ^a^	1.711 ± 0.025 ^c^
Hydroxyproline (Hyp)	0.051 ± 0.000 ^c^	0.053 ± 0.000 ^c^	0.065 ± 0.022 ^c^	0.088 ± 0.011 ^b^	0.212 ± 0.001 ^a^
Serine (Ser)	2.725 ± 0.001 ^c^	2.185 ± 0.008 ^d^	1.922 ± 0.001 ^e^	3.457 ± 0.048 ^a^	2.893 ± 0.002 ^b^
Asparagine (Asn)	2.405 ± 0.004 ^c^	2.095 ± 0.010 ^d^	1.992 ± 0.000 ^e^	3.406 ± 0.048 ^a^	2.785 ± 0.003 ^b^
Glycine (Gly)	1.479 ± 0.002 ^c^	1.127 ± 0.008 ^e^	1.289 ± 0.001 ^d^	1.713 ± 0.023 ^a^	1.521 ± 0.003 ^b^
Glutamine (Gln)	0.545 ± 0.001 ^e^	0.925 ± 0.003 ^d^	1.164 ± 0.000 ^c^	1.794 ± 0.027 ^a^	1.465 ± 0.001 ^b^
β-Alanine (βAla)	0.047 ± 0.000 ^d^	0.050 ± 0.002 ^c^	0.046 ± 0.000 ^c^	0.072 ± 0.001 ^a^	0.081 ± 0.000 ^b^
Taurine (Tau)	0.028 ± 0.001 ^c^	0.026 ± 0.000 ^d^	0.028 ± 0.000 ^c^	0.031 ± 0.001 ^b^	0.035 ± 0.000 ^a^
Histidine (His)	3.805 ± 0.058 ^c^	3.214 ± 0.008 ^e^	3.506 ± 0.002 ^d^	5.410 ± 0.010 ^a^	4.629 ± 0.029 ^b^
γ-Aminobutyric Acid (γaba)	0.069 ± 0.012 ^a^	0.004 ± 0.001 ^b^	0.025 ± 0.002 ^b^	0.064 ± 0.011 ^a^	0.048 ± 0.022 ^a^
Threonine (Thr)	1.790 ± 0.015 ^c^	1.507 ± 0.012 ^d^	1.542 ± 0.005 ^d^	3.243 ± 0.047 ^a^	2.136 ± 0.057 ^b^
Alanine (Ala)	4.319 ± 0.009 ^c^	3.543 ± 0.16 ^e^	3.991 ± 0.001 ^d^	6.110 ± 0.087 ^a^	5.241 ± 0.051 ^b^
Arginine (Arg)	1.734 ± 0.004 ^c^	1.674 ± 0.010 ^d^	1.780 ± 0.003 ^c^	3.452 ± 0.045 ^a^	2.381 ± 0.054 ^b^
Proline (Pro)	0.561 ± 0.002 ^d^	0.577 ± 0.006 ^c^	0.585 ± 0.004 ^c^	1.020 ± 0.014 ^a^	0.680 ± 0.003 ^a^
Tirosine (Tyr)	2.127 ± 0.002 ^a^	1.451 ± 0.008 ^d^	1.518 ± 0.002 ^c^	1.528 ± 0.025 ^c^	1.923 ± 0.031 ^b^
Valine (Val)	4.918 ± 0.007 ^c^	4.348 ± 0.007 ^d^	4.418 ± 0.010 ^d^	7.996 ± 0.137 ^a^	5.959 ± 0.015 ^b^
Metionine (Met)	0.769 ± 0.001 ^c^	0.646 ± 0.005 ^e^	0.705 ± 0.000 ^d^	0.900 ± 0.013 ^a^	0.824 ± 0.001 ^b^
Isoleucine (Ile)	0.429 ± 0.001 ^c^	0.374 ± 0.006 ^d^	0.278 ± 0.000 ^e^	0.920 ± 0.014 ^a^	0.538 ± 0.000 ^b^
Leucine (Leu)	8.693 ± 0.005 ^c^	7.332 ± 0.018 ^d^	7.033 ± 0.000 ^e^	12.165 ± 0.176 ^a^	9.035 ± 0.004 ^b^
Phenylalanine (Phe)	3.800 ± 0.002 ^c^	3.299 ± 0.009 ^d^	3.191 ± 0.001 ^e^	5.701 ± 0.087 ^a^	4.241 ± 0.002 ^b^
Tryptophan (Trp)	1.283 ± 0.002 ^b^	1.006 ± 0.018 ^d^	0.910 ± 0.001 ^e^	1.508 ± 0.024 ^a^	1.155 ± 0.001 ^c^
Ornitine (Orn)	0.111 ± 0.000 ^a^	0.046 ± 0.009 ^b^	0.045 ± 0.000 ^b^	0.045 ± 0.000 ^b^	0.051 ± 0.000 ^b^
Lysine (Lys)	6.403 ± 0.002 ^c^	5.677 ± 0.018 ^e^	6.057 ± 0.001 ^d^	8.851 ± 0.130 ^a^	7.686 ± 0.001 ^b^
Total	52.291 ± 0.110 ^c^	44.908 ± 0.089 ^e^	45.170 ± 1.151 ^d^	74.286 ± 1.113 ^a^	59.628 ± 0.205 ^b^

**Table 2:** In silico characterization of LC-MS/MS-identified peptides in porcine blood hydrolysates with peptide ranker ratio (PRR) > 0.75.

Sample	Peptide Sequence	PRR ^a^	Allergenicity	FRS Score ^b^	Chel. Score ^b^	Hydrophobicity	Toxicity	Net Charge	CPPpred
APP2	DRPFPDF	0.9478	non-allergen	0.4197	0.2804	−0.3	non-toxic	−1	0.0751
	QPFPIRP	0.9270	allergen	0.4315	0.2672	−0.19	non-toxic	1	0.1219
	DGPLPRP	0.8566	non-allergen	0.4772	0.2914	−0.29	non-toxic	0	0.2438
	LFPNPPPPPQP	0.8564	non-allergen	0.6587	0.3414	−0.06	non-toxic	0	0.1297
	ADPVNFPKL	0.8482	non-allergen	0.4004	0.2633	−0.07	non-toxic	0	0.1646
	ASQPDVDGFLVGGASLKPEF	0.8193	non-allergen	0.4769	0.2594	−0.01	non-toxic	−2	0.1123
	GPGGPGTWKPGRPEPG	0.802	allergen	0.5683	0.1886	−0.17	non-toxic	1	0.2960
	SMGKGYYLKGKIGKVPVRF	0.7891	allergen	0.4137	0.1610	−0.14	non-toxic	5	0.2669
	DPQSPWDR	0.7839	allergen	0.4375	0.2600	−0.49	non-toxic	−1	0.1224
	GPGSVPGTGSPGGLKPG	0.7815	non-allergen	0.4537	0.2112	0.01	non-toxic	1	0.1529
	DPENFRL	0.7746	allergen	0.3757	0.2620	−0.38	non-toxic	−1	0.1295
	YQEPVLGPVRGPFPIIV	0.7744	allergen	0.5392	0.1770	0.07	non-toxic	0	0.1219
	AAGPPISEGKYF	0.7593	non-allergen	0.4157	0.2176	0	non-toxic	0	0.0736
	GPGSVPGTGSPGGLKP	0.7557	non-allergen	0.4383	0.2098	0	non-toxic	1	0.1546
	VDGPLPRP	0.7519	non-allergen	0.4872	0.2716	−0.18	non-toxic	0	0.2618
	HPDDFNP	0.7510	allergen	0.3928	0.2717	−0.29	non-toxic	−1.5	0.0494
APP6	GYFVFGPTGCNLEGFF	0.9328	non-allergen	0.4997	0.2137	0.17	non-toxic	−1	0.0618
	DPQSPWDR	0.7839	allergen	0.4375	0.26000	−0.49	non-toxic	−1	0.1224
	YQEPVLGPVRGPFPIIV	0.7744	allergen	0.5392	0.1770	0.07	non-toxic	0	0.1219
APP24	AAEGACQQCSCRPMWCLTCMGKWFASRQDPQRPDTWL	0.9733	non-allergen	-	-	−0.2	toxic	1	0.4553
	QPFPIRP	0.9270	allergen	0.4315	0.2672	−0.19	non-toxic	1	0.1219
	DPQSPWDR	0.7839	allergen	0.4375	0.2600	−0.49	non-toxic	−1	0.1224
	YQEPVLGPVRGPFPIIV	0.7744	allergen	0.5392	0.1770	0.07	non-toxic	0	0.1219
	GPGSVPGTGSPGGLKP	0.7557	non-allergen	0.4383	0.2098	0	non-toxic	1	0.1546
	HPDDFNP	0.7510	allergen	0.3928	0.2717	−0.29	non-toxic	−1.5	0.0494
AFPP	GGGFGGGGGIGGGGGGGGGGGG	0.8075	non-allergen	0.4887	0.1860	0.21	non-toxic	0	0.1023
	DPQSPWDR	0.7839	allergen	0.4375	0.2600	−0.49	non-toxic	−1	0.1224
	YQEPVLGPVRGPFPIIV	0.7744	allergen	0.5392	0.1770	0.07	non-toxic	0	0.1219
	DDPQSPWDR	0.7303	non-allergen	0.3971	0.2742	−0.52	non-toxic	−2	0.1006
APPP	DPQSPWDR	0.7839	allergen	0.4375	0.2600	−0.49	non-toxic	−1	0.1224
	DPENFRL	0.7746	allergen	0.3757	0.2620	−0.38	non-toxic	−1	0.1295
	YQEPVLGPVRGPFPIIV	0.7744	allergen	0.5392	0.1770	0.07	non-toxic	0	0.1219
	HPDDFNP	0.7510	allergen	0.3928	0.2717	−0.29	non-toxic	−1.5	0.0494

^a^ PRR: peptide ranker ratio. ^b^ Antioxidant properties prediction according to AnOxPePred tool. FRS: free radical scavenging capacity. Chel.: chelating activity.

**Table 3:** Simulated gastrointestinal digestion of peptides with PRR > 0.75, previously identified via LC-MS/MS of porcine blood hydrolysates.

Sample	Peptide Sequence	PRR	GID-Enzymes Action	Active Fragment	Bioactivity of the Fragment	CPP Fragment
APP2	DRPFPDF	0.9478	DR–PF–PDF	DR	ACE inhibitor	0.4317
					DPP IV inhibitor	
				PF	ACE inhibitor	0.0466
					DPP IV inhibitor	
					DPP-III inhibitor	
	QPFPIRP	0.927	QPF–PIR–P	-	-	-
	DGPLPRP	0.8566	DGPL–PR–P	PR	ACE inhibitor	0.8096
					DPP-III inhibitor	
	LFPNPPPPPQP	0.8564	L–F–PN–PPPPPQP	PN	DPP IV inhibitor	0.1469
	ADPVNFPKL	0.8482	ADPVN–F–PK–L	PK	Antioxidative	0.5177
					DPP IV inhibitor	
	ASQPDVDGFLVGGASLKPEF	0.8193	ASQPDVDGF–L–VGGASL–K–PEF	-	-	-
	GPGGPGTWKPGRPEPG	0.8020	GPGGPGTW–K–PGR–PEPG	-	-	-
	SMGKGYYLKGKIGKVPVR	0.7891	SM–GK–GY–Y–L–K–GK–IGK–VPVR–F	GK	ACE inhibitor	0.5298
				GY	ACE inhibitor	0.0443
					Inhibitor of tripeptidyl peptidase II	
					DPP IV inhibitor	
				SM	DPP-III inhibitor	0.0482
	DPQSPWDR	0.7839	DPQSPW–DR-	DR	ACE inhibitor	0.4317
					DPP IV inhibitor	
	GPGSVPGTGSPGGLKPG	0.7815	GPGSVPGTGSPGGL–K–PG	-	-	-
	DPENFRL	0.7746		-	-	-
	YQEPVLGPVRGPFPIIV	0.7744	Y–QEPVL–GPVR–GPF–PIIV	GPF	DPP IV inhibitor	0.0477
	AAGPPISEGKYF	0.7593	AAGPPISEGK–Y–F-	-	-	
	GPGSVPGTGSPGGLKP	0.7557	GPGSVPGTGSPGGL–K–P	-	-	
	VDGPLPRP	0.7519	VDGPL–PR–P	PR	ACE inhibitor	0.8096
					DPP-III inhibitor	
	HPDDFNP	0.7510	H–PDDF–N–P	-	-	-
APP6	GYFVFGPTGCNLEGFF	0.9328	GY–F–VF–GPTGCN–L–EGF–F	GY	ACE inhibitor	0.0443
					DPP IV inhibitor	
					Inhibitor of tripeptidyl peptidase II	
				VF	ACE inhibitor	0.0751
					DPP IV inhibitor	
					Calpain 1 inhibitor	
					Inhibitor of tripeptidyl peptidase II	
					Inhibitor of neprilysin-2	
	DPQSPWDR	0.7839	DPQSPW–DR	DR	ACE inhibitor	0.4317
					DPP IV inhibitor	
	YQEPVLGPVRGPFPIIV	0.7744	Y–QEPVL–GPVR–GPF–PIIV	GPF	DPP IV inhibitor	0.0477
APP24	AAEGACQQCSCRPMWCLTCMGKWFASRQDPQRPDTWL	0.9733	AAEGACQQCSCR–PM–W–CL–TCM–GK–W–F–ASR–QDPQR–PDTW–L-	GK	ACE inhibitor	0.5298
				PM	ACE inhibitor	0.1096
					DPP IV inhibitor	
	QPFPIRP	0.9270	QPF–PIR–P	-	-	-
	DPQSPWDR	0.7839	DPQSPW–DR-	DR	ACE inhibitor	0.4317
					DPP IV inhibitor	
	YQEPVLGPVRGPFPIIV	0.7744	Y–QEPVL–GPVR–GPF–PIIV	GPF	DPP IV inhibitor	0.0477
	GPGSVPGTGSPGGLKP	0.7557	GPGSVPGTGSPGGL–K–P	-	-	
	HPDDFNP	0.7510	H–PDDF–N–P	-	-	-
AFPP	GGGFGGGGGIGGGGGGGGGGGG	0.8075	GGGF–GGGGGIGGGGGGGGGGGG	-	-	-
	DPQSPWDR	0.7839	DPQSPW–DR-	DR	ACE inhibitor	0.4317
					DPP IV inhibitor	
	YQEPVLGPVRGPFPIIV	0.7744	Y–QEPVL–GPVR–GPF–PIIV	GPF	DPP IV inhibitor	0.0477
	DDPQSPWDR	0.7303	DDPQSPW–DR-	DR	ACE inhibitor	0.4317
					DPP IV inhibitor	
APPP	DPQSPWDR	0.7839	DPQSPW–DR	DR	ACE inhibitor	0.4317
					DPP IV inhibitor	
	DPENFRL	0.7746	DPEN–F–R–L-	-	-	-
	YQEPVLGPVRGPFPIIV	0.7744	Y–QEPVL–GPVR–GPF–PIIV	GPF	DPP IV inhibitor	0.0477
	HPDDFNP	0.7510	H–PDDF–N–P	-	-	-

## Data Availability

Data contained within the article.
